# Supplementary material for: Quantifying synthetic bacterial community composition with flow cytometry: efficacy in mock communities and challenges in co-cultures
Source: mSystems. 2024 Nov 29;10(1):e01009-24. doi: 10.1128/msystems.01009-24 (PMC11748490; doi:10.1128/msystems.01009-24)
Supplement: Supplemental material — Supplemental figures and tables. [file msystems.01009-24-s0001.pdf]

Supplementary information for

Quantifying synthetic bacterial community composition with flow cytometry: efficacy in mock communities and challenges in co-cultures

Authors

Fabian Mermans[a,b], Ioanna Chatzigiannidou[a], Wim Teughels[b], Nico Boon[a#]

Affiliation

[a] Center for Microbial Ecology and Technology (CMET), Faculty of Bioscience Engineering, Ghent University, Frieda Saeysstraat 1, B-9052 Gent, Belgium

[b] Department of Oral Health Sciences, KU Leuven & Dentistry (Periodontology), University Hospitals Leuven, Kapucijnenvoer 7, B-3000 Leuven, Belgium.

[#] Correspondence to: Nico Boon, Ghent University, Faculty of Bioscience Engineering; Center of Microbial Ecology and Technology (CMET); Frieda Saeysstraat 1, B-9052 Gent, Belgium; phone: +32 (0)9 264 59 76; fax: +32 (0)9 264 62 48; E-mail: Nico.Boon@UGent.be

Supplementary 1. Composition of mock communities

The species used in the mock communities, and by extension in the co-cultures, are naturally occurring in the oral cavity. *A. actinomycetemcomitans*, *P. gingivalis* and *P. intermedia* are known to be involved in periodontal disease, while *S. mutans* and *S. sobrinus* are key organisms in dental caries (1–5). *F. nucleatum* and the *Actinomyces* species are considered important bridging organisms for oral biofilm development (6–8). Finally, the remaining bacteria are commensal oral streptococci that are primary colonizers in the oral cavity and play an important role in maintaining host-microbiome homeostasis (5).

Table S1. Composition of mock communities.

**Mock community**	**Strain**	**Replicate axenic culture**	**Volume (µL)**
Mock 1	*F. nucleatum*	A	810
	S. oralis	A	810
Mock 2	*F. nucleatum*	A	400
	P. gingivalis	A	400
	S. oralis	A	400
Mock 3	*F. nucleatum*	B	300
	P. gingivalis	A	300
	S. oralis	B	300
	V. parvula	A	300
Mock 4	*A. naeslundii*	A	500
	A. viscosus	A	150
	S. gordonii	A	150
	S. mitis	A	75
	S. oralis	B	150
	S. salivarius	A	150
	S. sanguinis	A	75
	V. parvula	A	150
Mock 5	*S. gordonii*	A	200
	S. mitis	A	100
	S. mutans	A	200
	S. oralis	B	200
	S. salivarius	A	200

	S. sanguinis	A	100
	S. sobrinus	A	200
Mock 6	*A. actinomycetemcomitans*	A	50
	F. nucleatum	B	250
	P. gingivalis	A	250
	P. intermedia	A	250
	S. mutans	A	250
	S. sobrinus	A	250
Mock 7	*A. actinomycetemcomitans*	A	20
	A. naeslundii	A	100
	A. viscosus	A	100
	F. nucleatum	B	100
	P. gingivalis	A	100
	P. intermedia	A	100
	S. gordonii	A	100
	S. mitis	A	50
	S. mutans	A	100
	S. oralis	B	100
	S. salivarius	A	100
	S. sanguinis	A	50
	S. sobrinus	A	100
	V. parvula	A	100
Mock 8	*F. nucleatum*	A	10
	S. oralis	C	1000
Mock 9	*F. nucleatum*	C	1000
	S. oralis	C	10

Supplementary 2. Strain specific primers and probes for qPCR

Table S2. List of strain specific primers and probes for qPCR.

Strain	Target gene	Copy number	Sequence (5' → 3')	
Aggregatibacter actinomycetemcomitans	16S rRNA	6	Forward	GAA CCT TAC CTA CTC TTG ACA TCC GAA
			Reverse	TGC AGC ACC TGT CTC AAA GC
			Probe	AGA ACT CAG AGA TGG GTT TGT GCC TTA GGG
Actinomyces naeslundii	Unknown	3	Forward	TCG AAA CTC AGC AAG TAG CCG
			Reverse	AGA GGA GGG CCA CAA AAG AAA
			Probe	GGG TAC TCT AGT CCA AAC TGG CGG ATA GCG
Actinomyces viscosus	16S rRNA	3	Forward	GTG AAG GAG CCA GCT TGC TGG TTC TG
			Reverse	CGG AAC AAA CCT TTC CCA GGC
			Probe	ATG AGT GGC GAA CGG GTG AGT AAC
Fusobacterium nucleatum	16S rRNA	5	Forward	GGA TTT ATT GGG CGT AAA GC
			Reverse	GGC ATT CCT ACA AAT ATC TAC GAA
			Probe	CTC TAC ACT TGT AGT TCC G
Porphyromonas gingivalis	16S rRNA	4	Forward	GCG CTC AAC GTT CAG CC
			Reverse	CAC GAA TTC CGC CTG C
			Probe	CAC TGA ACT CAA GCC CGG CAG TTT CAA
Prevotella intermedia	16S rRNA	4	Forward	CGG TCT GTT AAG CGT GTT GTG
			Reverse	CAC CAT GAA TTC CGC ATA CG
			Probe	TGG CGG ACT TGA GTG CAC GC

Streptococcus gordonii	gftG	1	Forward	CGG ATG ATG CTA ATC AAG TGA CC
			Reverse	GTT AGC TGT TGG ATT GGT TGC C
			Probe	AGA ACA GTC CGC TGT TCA GAG CAA
Streptococcus mitis	16S rRNA	3	Forward	GGC TCG TAG TCT GGA GAT GG
			Reverse	TAG GTC GTC GTC CCA AGG AA
			Probe	CGA AGA GCA CCA ATA GCA CCT CCC
Streptococcus mutans	gftB	1	Forward	GCC TAC AGC TCA GAG ATG CTA TTC T
			Reverse	GCC ATA CAC CAC TCA TGA ATT GA
			Probe	TGG AAA TGA CGG TCG CCG TTA TGA A
Streptococcus oralis	gftR	1	Forward	ACC AGC AGA TAC GAA AGA AGC AT
			Reverse	AGG TTC GGG CAA GCG ATC TTT CT
			Probe	AAG GCT GCT GTT GCT GAA GAA GT
Streptococcus salivarius	dextranese	1	Forward	AAC GTT GAC CTT ACG CTA GC
			Reverse	ACC GTA ACG TGG GAA AAC TG
			Probe	GTA GCG TCA GAG TGG TTG AC
Streptococcus sanguinis	gftP	1	Forward	CAA AAT TGT TGC AAA TCC AAA GG
			Reverse	GCT ATC GCT CCC TGT CTT TGA
			Probe	AAA GAA AGA TCG CTT GCC AGA ACC GG
Streptococcus sobrinus	gftT	1	Forward	TTC AAA GCC AAG ACC AAG CTA GT
			Reverse	CCA GCC TGA GAT TCA GCT TGT
			Probe	CCT GCT CCA GCG ACA AAG GCA GC

Veillonella parvula	16S rRNA	4	Forward	GAC GAA AGT CTG ACG GAG CA
			Reverse	TGC CAC CTA CGT ATT ACC GC
			Probe	AGC TCT GTT AAT CGG GAC GAA AGG C

Supplementary 3. Gating strategy

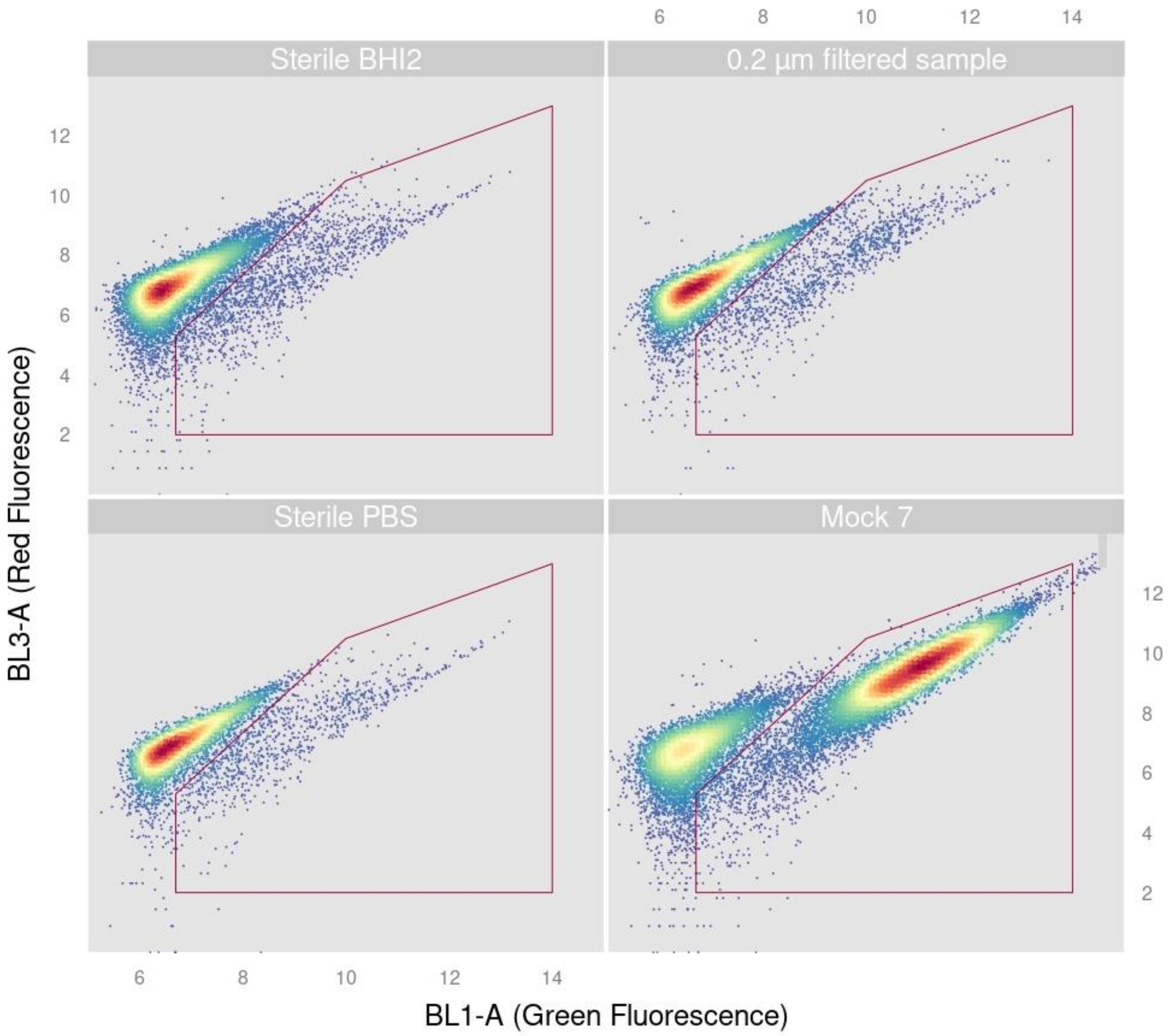

Figure S1. Gating strategy for gating bacterial cells. Negative controls are represented by 'Sterile BHI2' (growth medium), '0.2 µm filtered sample' (bacterial culture that was filtered through a 0.2 µm filter), and 'Sterile PBS' (dilutant used for bacterial cultures). *Mock 7* contained all bacterial strains used in the experiment. The gate was drawn so that bacterial cells were included, and background signal was excluded.

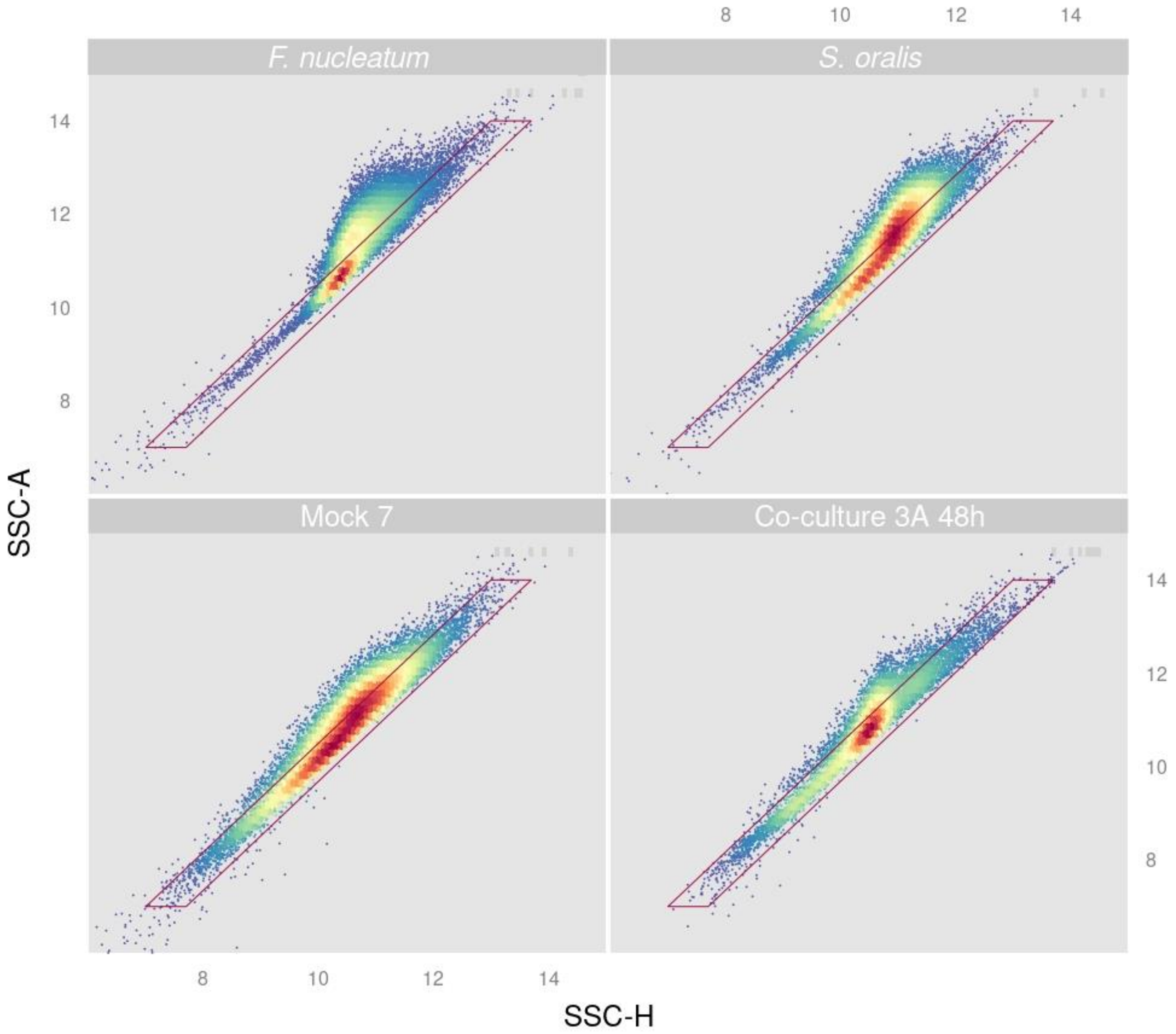

Figure S2. Gating strategy for gating singlets. Gating of singlets was done by assessing the relationship between peak height (SSC-H) and peak area (SSC-A) of side scatter. As an example, two bacterial strains (*F. nucleatum* and *S. oralis*), *Mock 7* and *Co-culture 3* (replicate A) after 48h of growth were included in the figure. *Mock 7* contained all bacterial strains used in the experiment, while *Co-culture 3* was the most complex co-culture in the experiment. The gate was drawn so that singlets were included and multiplets were excluded.

Supplementary 4. Random forest models

Table S3. List of random forest models trained on flow cytometry data from axenic cultures.

Name model	Included strains	Number of strains
FCM SoFn	*F. nucleatum*<br>*S. oralis*	2
FCM SoFnPg	*F. nucleatum*<br>*P. gingivalis*<br>*S. oralis*	3
FCM SoFnPgVp	*F. nucleatum*<br>*P. gingivalis*<br>*S. oralis*<br>*V. parvula*	4
FCM Commensals	*A. naeslundii*<br>*A. viscosus*<br>*S. gordonii*<br>*S. mitis*<br>*S. oralis*<br>*S. salivarius*<br>*S. sanguinis*<br>*V. parvula*	8
FCM Streps	*S. gingivalis*<br>*S. mitis*<br>*S. mutans*<br>*S. oralis*<br>*S. salivarius*<br>*S. sanguinis*<br>*S. sobrinus*	7
FCM Pathogens	*A. actinomycetemcomitans*<br>*F. nucleatum*<br>*P. gingivalis*<br>*P. intermedia*<br>*S. mutans*<br>*S. sobrinus*	6

FCM All	*A. actinomycetemcomitans*<br>*A. naeslundii*<br>*A. viscosus*<br>*F. nucleatum*<br>*P. gingivalis*<br>*P. intermedia*<br>*S. gordonii*<br>*S. mitis*<br>*S. mutans*<br>*S. oralis*<br>*S. salivarius*<br>*S. sanguinis*<br>*S. sobrinus*<br>*V. parvula*	14

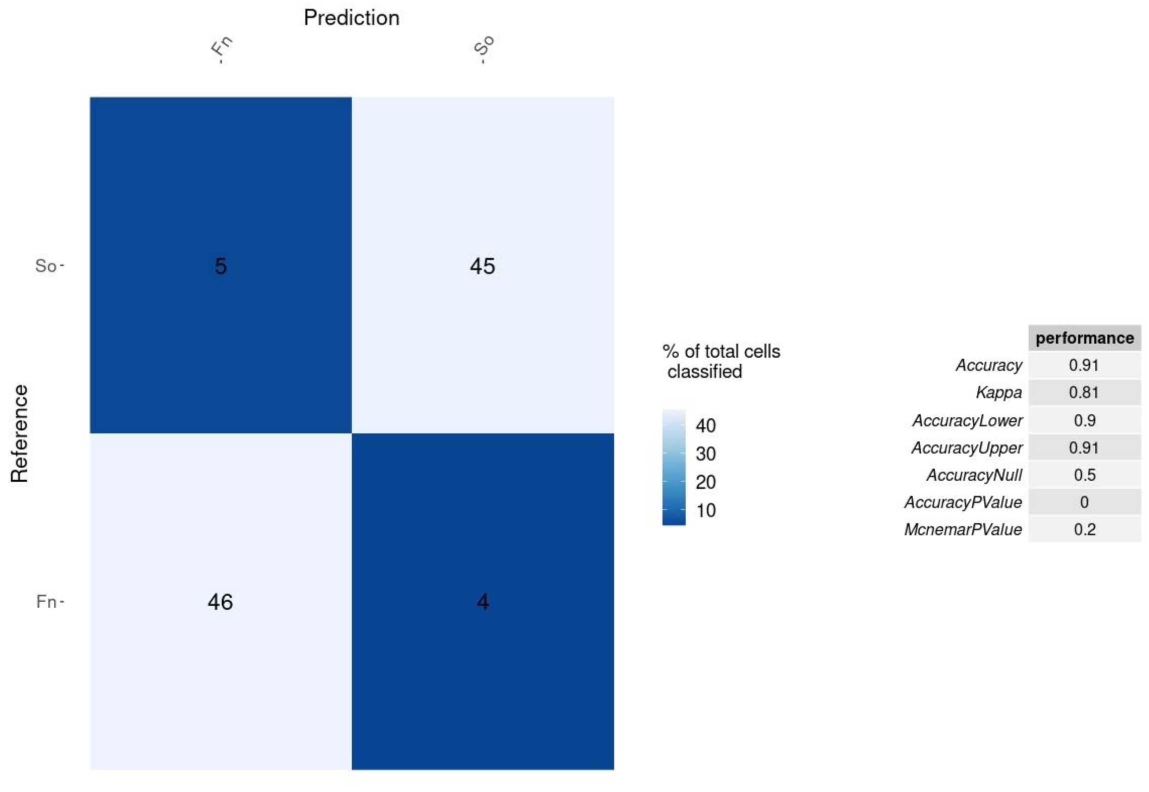

Figure S3. Confusion matrix for random forest model *FCM SoFn*. The model includes *S. oralis* (So) and *F. nucleatum* (Fn).

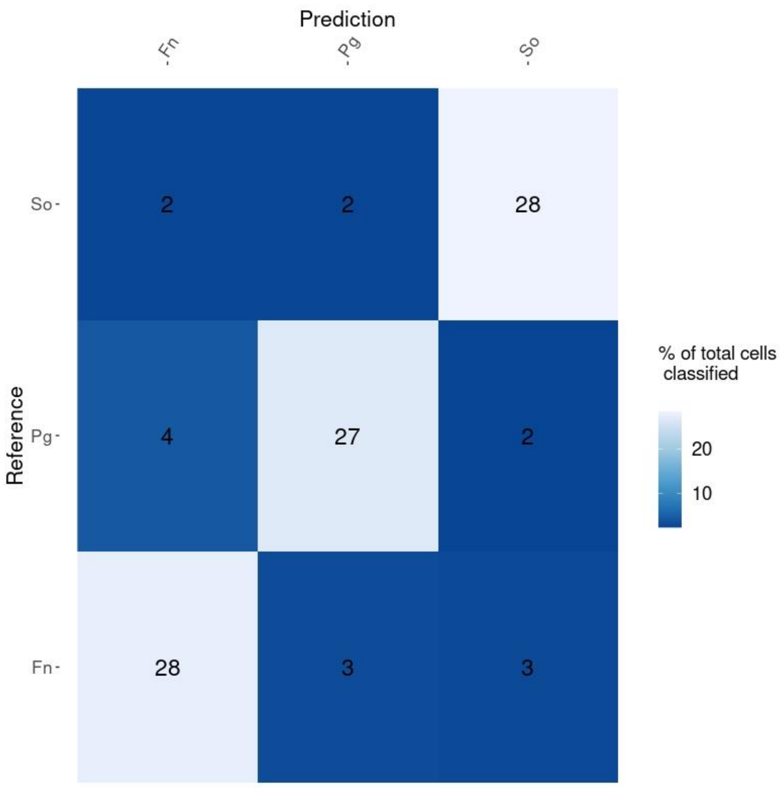

	performance
Accuracy	0.83
Kappa	0.74
AccuracyLower	0.82
AccuracyUpper	0.83
AccuracyNull	0.33
AccuracyPValue	0
McnemarPValue	0

Figure S4. Confusion matrix for random forest model *FCM SoFnPg*. The model includes *S. oralis* (So), *F. nucleatum* (Fn) and *P. gingivalis* (Pg).

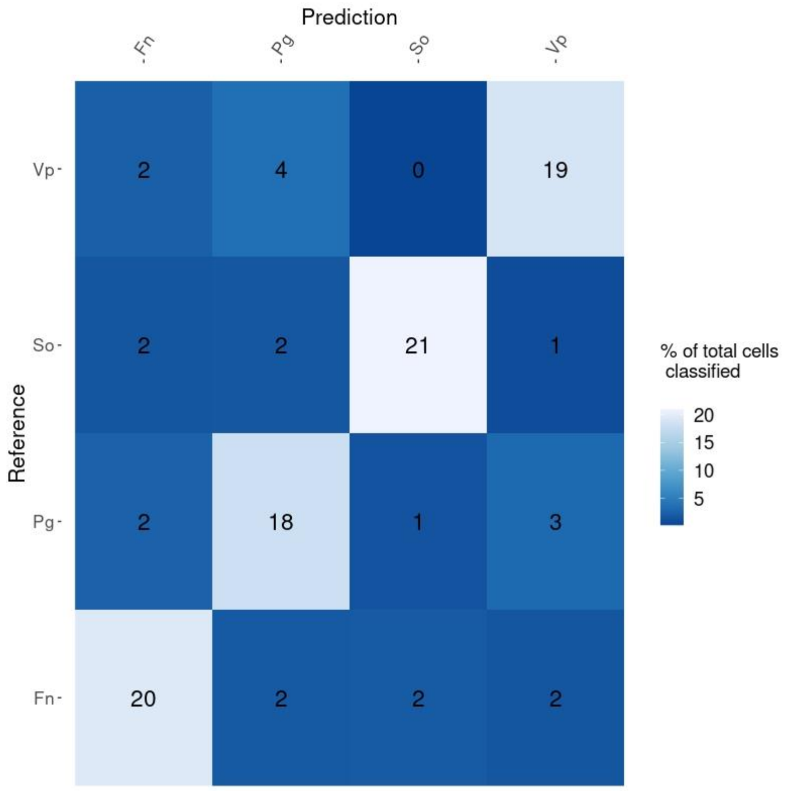

	performance
Accuracy	0.78
Kappa	0.7
AccuracyLower	0.77
AccuracyUpper	0.78
AccuracyNull	0.25
AccuracyPValue	0
McnemarPValue	0

Figure S5. Confusion matrix for random forest model *FCM SoFnPgVp*. The model includes *S. oralis* (So), *F. nucleatum* (Fn), *P. gingivalis* (Pg) and *V. parvula* (Vp).

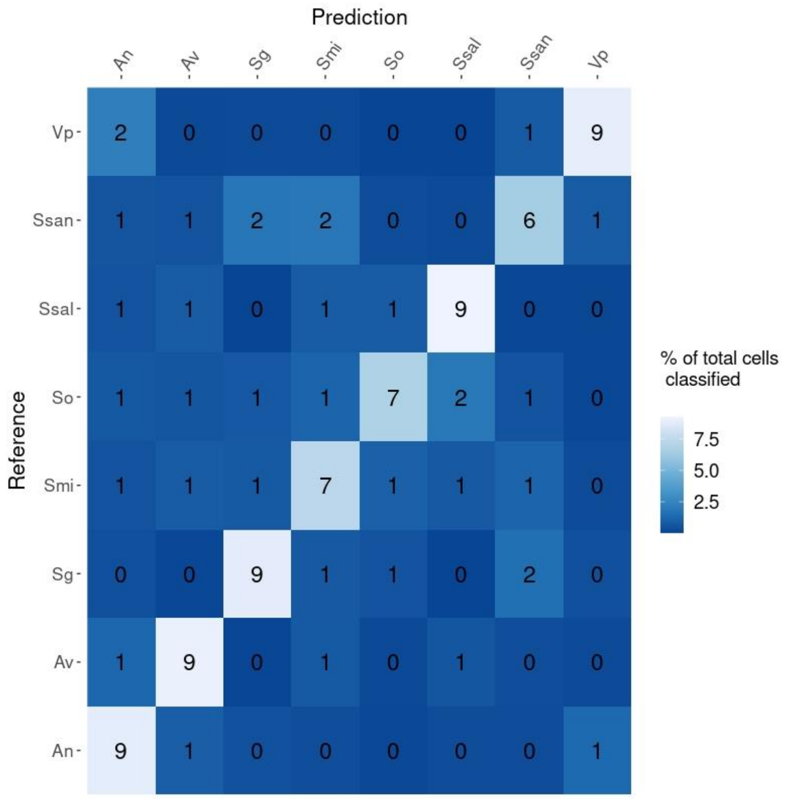

	performance
Accuracy	0.66
Kappa	0.61
AccuracyLower	0.65
AccuracyUpper	0.66
AccuracyNull	0.12
AccuracyPValue	0
McnemarPValue	0

Figure S6. Confusion matrix for random forest model *FCM Commensals*. The model includes *A. naeslundii* (An), *A. viscosus* (Av), *S. gordonii* (Sg), *S. mitis* (Smi), *S. oralis* (So), *S. salivarius* (Ssal), *S. sanguinis* (Ssan) and *V. parvula* (Vp).

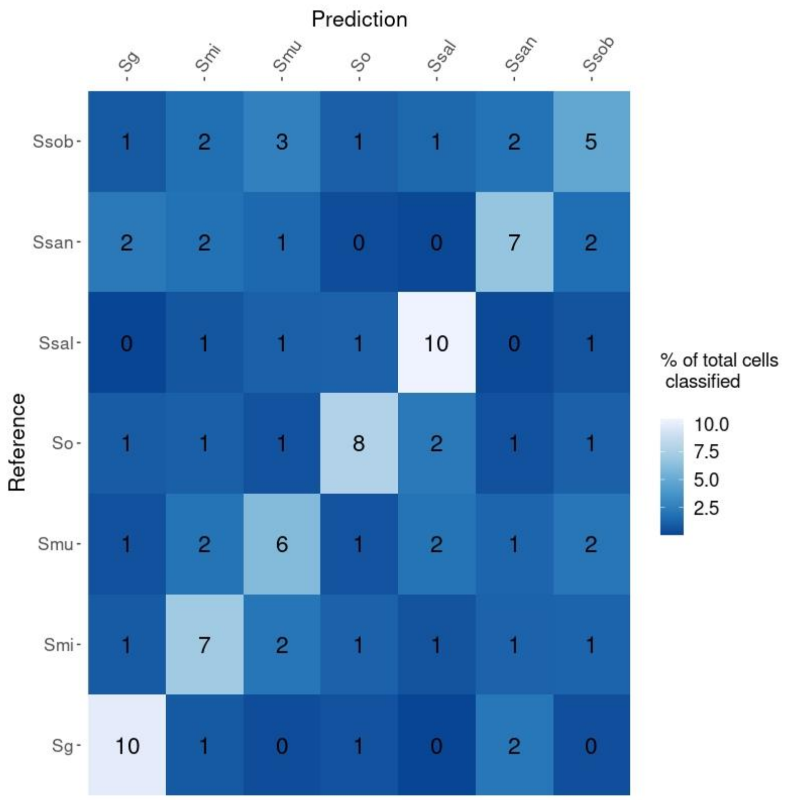

	performance
Accuracy	0.53
Kappa	0.45
AccuracyLower	0.52
AccuracyUpper	0.53
AccuracyNull	0.14
AccuracyPValue	0
McnemarPValue	0

Figure S7. Confusion matrix for random forest model *FCM Streps*. The model includes *S. gordonii* (Sg), *S. mitis* (Smi), *S. mutans* (Smu), *S. oralis* (So), *S. salivarius* (Ssal), *S. sanguinis* (Ssan) and *S. sobrinus* (Ssob).

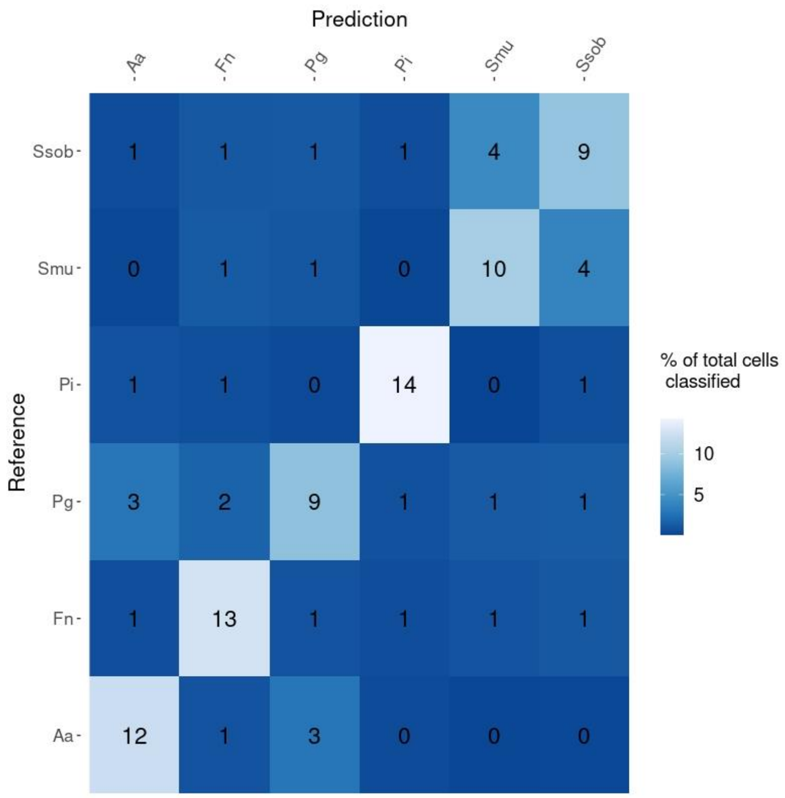

	performance
Accuracy	0.67
Kappa	0.6
AccuracyLower	0.66
AccuracyUpper	0.68
AccuracyNull	0.17
AccuracyPValue	0
McnemarPValue	0

Figure S8. Confusion matrix for random forest model *FCM Pathogens*. The model includes *A. actinomycetemcomitans* (Aa), *F. nucleatum* (Fn), *P. gingivalis* (Pg), *P. intermedia* (Pi), *S. mutans* (Smu) and *S. sobrinus* (Ssob).

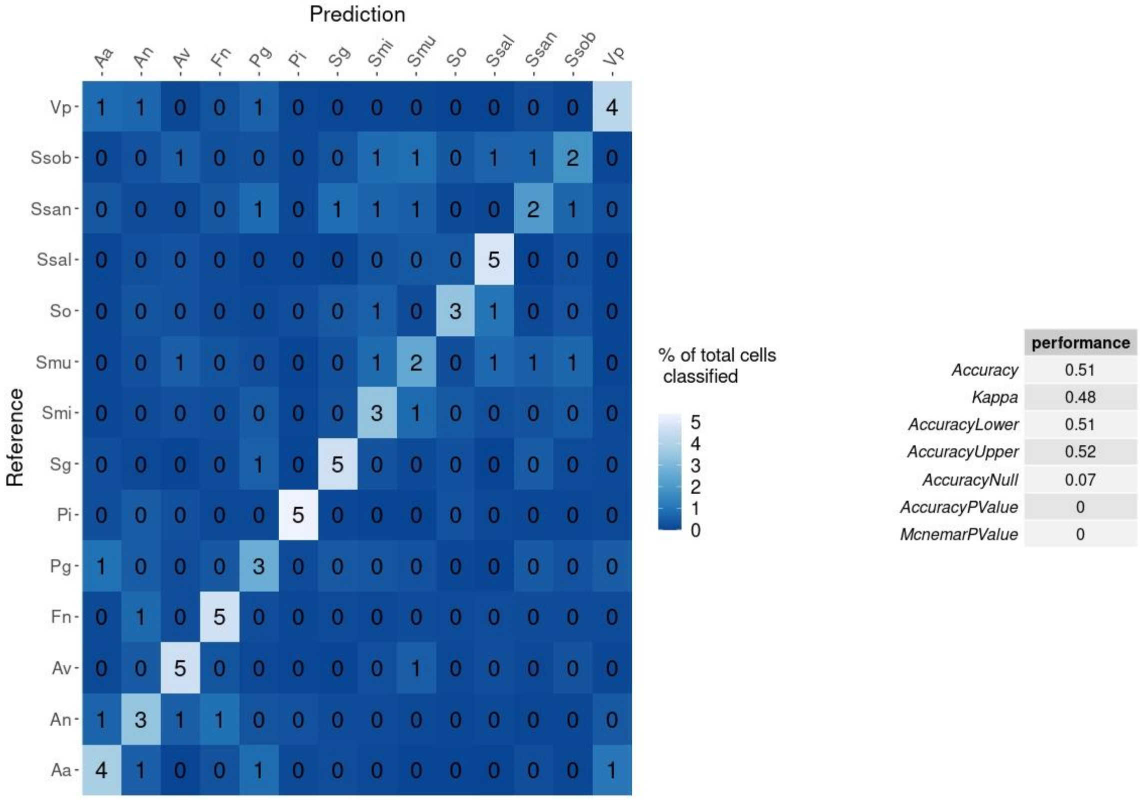

Figure S9. Confusion matrix for random forest model *FCM All*. The model includes *A. actinomycetemcomitans* (Aa), *A. naeslundii* (An), *A. viscosus* (Av), *F. nucleatum* (Fn), *P. gingivalis* (Pg), *P. intermedia* (Pi), *S. gordonii* (Sg), *S. mitis* (Smi), *S. mutans* (Smu), *S. oralis* (So), *S. salivarius* (Ssal), *S. sanguinis* (Ssan), *S. sobrinus* (Ssob) and *V. parvula* (Vp).

Supplementary 5. Singlet analysis

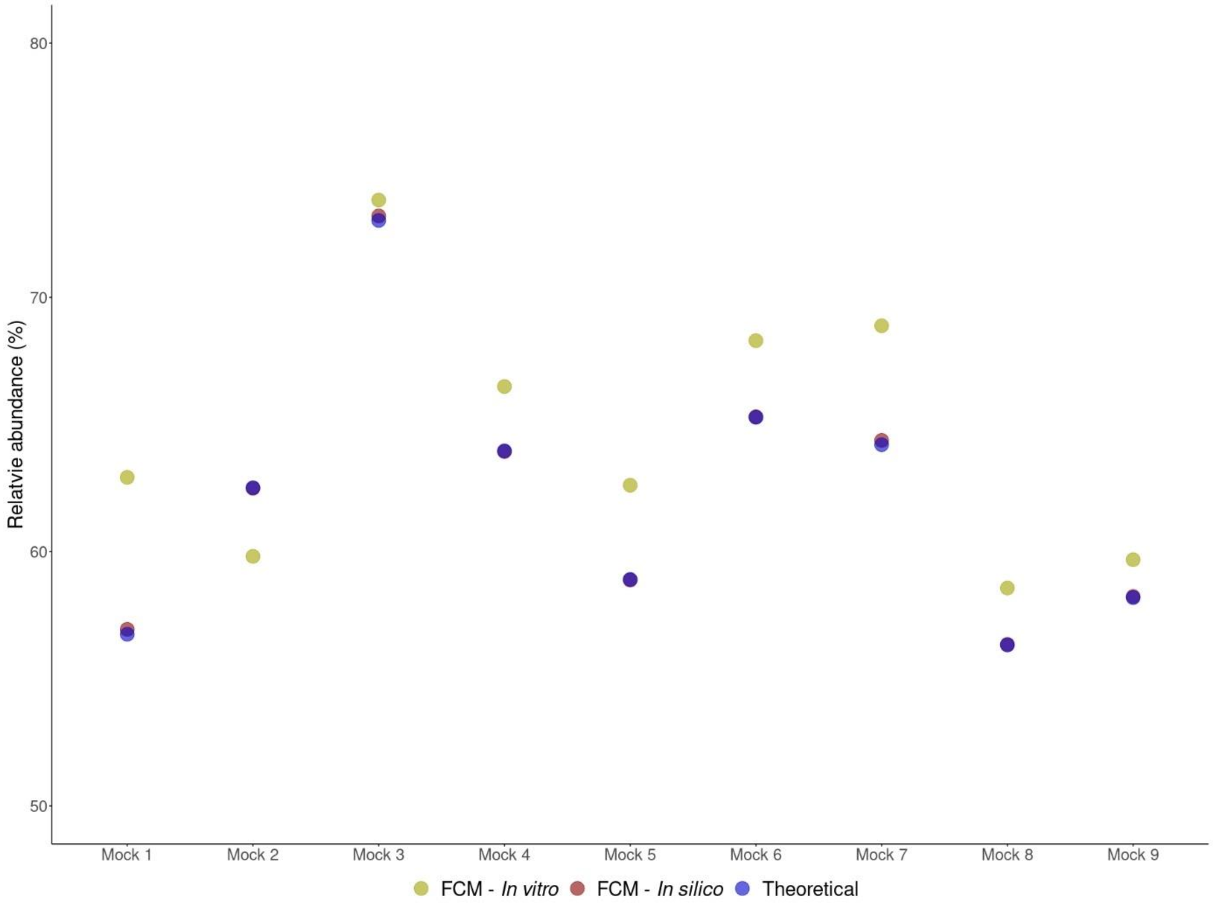

Figure S10. Relative abundance of singlets in mock communities. 'FCM – *In vitro*' refers to the mock communities which were prepared *in vitro*, 'FCM – *In silico*' refers to the mock communities prepared *in silico*, and 'Theoretical' refers to the theoretically calculated relative abundance of singlets based on the flow cytometry measurements of axenic cultures.

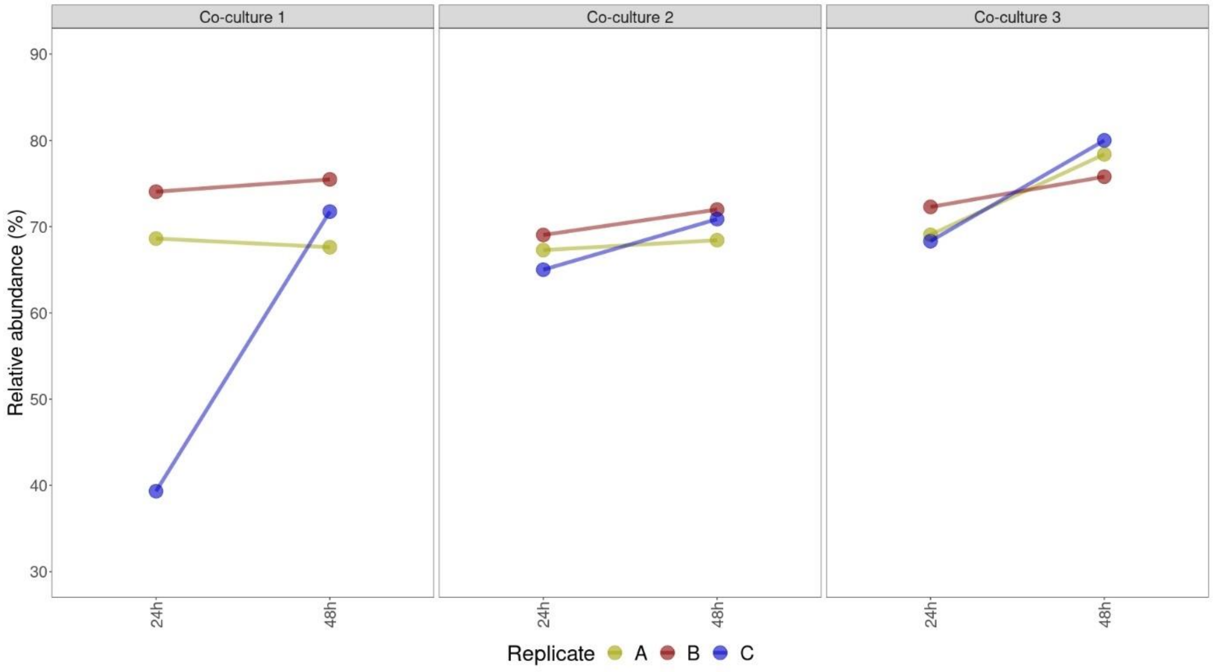

Figure S11. Relative abundance of singlets in co-cultures. The different colors represent the different replicates.

Supplementary 6. Statistical analysis cell concentrations co-cultures

Paired t-tests were performed to assess the difference in cell concentration between 24h of growth and 48h of growth of the co-cultures. Shapiro-Wilk's method and visual inspection of Q-Q plots were used to check the underlying assumption of normality. For all co-cultures the assumption was met.

Table S4. Statistical analysis of difference in cell concentration between timepoints for the co-cultures according to qPCR.

Co-culture	Two-sided paired t-test	
	t	p
Co-culture 1	8.4734	0.01364
Co-culture 2	-2.7464	0.1109
Co-culture 3	1.3883	0.2995

Table S5. Statistical analysis of difference in cell concentration between timepoints for the co-cultures according to flow cytometry.

Co-culture	Two-sided paired t-test	
	t	p
Co-culture 1	-0.21851	0.8473
Co-culture 2	0.73369	0.5395
Co-culture 3	-3.2085	0.08495

References

1. Marsh PD. 2018. In Sickness and in Health - What Does the Oral Microbiome Mean to Us? An Ecological Perspective. Adv Dent Res 29:60–65.
2. Lemos JA, Palmer SR, Zeng L, Wen ZT, Kajfasz JK, Freires IA, Abranches J, Brady LJ. 2019. The Biology of Streptococcus mutans. Microbiol Spectr 7.
3. Lamont RJ, Hajishengallis G. 2015. Polymicrobial synergy and dysbiosis in inflammatory disease. Trends Mol Med 21:172–183.
4. Lamont RJ, Koo H, Hajishengallis G. 2018. The oral microbiota: dynamic communities and host interactions. Nat Rev Microbiol 16:745–759.
5. Rosier BT, Marsh PD, Mira A. 2018. Resilience of the Oral Microbiota in Health: Mechanisms That Prevent Dysbiosis. J Dent Res 97:371–380.
6. Kolenbrander PE, Palmer RJ, Periasamy S, Jakubovics NS. 2010. Oral multispecies biofilm development and the key role of cell–cell distance. Nat Rev Microbiol 8:471–480.
7. Akbar P, Salahuddin N, Ahmad Z, Shah SZ, Shah F, Maknoon D. 2024. Role of Actinomyces species in oral Biofilm Formation and Dental Plaque-Related Diseases. Innovative Research in Applied, Biological and Chemical Sciences 2:120–125.
8. Dige I, Raarup MK, Nyengaard JR, Kilian M, Nyvad B. 2009. Actinomyces naeslundii in initial dental biofilm formation. Microbiology (N Y) 155:2116–2126.
